# Does antibiotic resistance impair plasma susceptibility of multi-drug resistant clinical isolates of enterococci in vitro?

**DOI:** 10.1186/s13099-016-0122-4

**Published:** 2016-09-01

**Authors:** Matthias Napp, Sebastian von Podewils, Ingo Klare, Hermann Haase, Richard Kasch, Denis Gümbel, Axel Ekkernkamp, Michael Jünger, Georg Daeschlein

**Affiliations:** 1 Department of Surgery, Ernst Moritz Arndt University, Sauerbruchstrasse, 17475 Greifswald, Germany; 2 Department of Dermatology, Ernst Moritz Arndt University, Sauerbruchstrasse, 17475 Greifswald, Germany; 3 National Reference Center for Staphylococci and Enterococci, Robert-Koch-Institute, Wernigerode branch, Burgstraße 37, 38855 Wernigerode, Germany; 4 Department of Orthopaedics, Ernst Moritz Arndt University, Sauerbruchstrasse, 17475 Greifswald, Germany; 5 Unfallkrankenhaus Berlin (ukb), Warener Straße 7, 12683 Berlin, Germany

**Keywords:** Dielectric barrier discharge plasma (DBD), Cold atmospheric plasma (CAP), Antimicrobial efficacy, Plasma susceptibility, Plasma medicine, Vancomycin-resistant enterococci, Gentamicin-resistant enterococci

## Abstract

**Background:**

Cold atmospheric plasma could constitute an alternative against multi-drug resistant pathogens. Susceptibility of enterococci to cold atmospheric plasma was investigated in vitro.

**Methods:**

39 clinical isolates of enterococci were grouped dependent on the most important resistance patterns and treated on agar using dielectric barrier discharge plasma. These included enterococci with combined vancomycin- and high-level gentamicin resistance, high-level resistance to gentamicin (HLGR) only, vancomycin resistance alone (VRE), and enterococci susceptible to both. Susceptibility to cold atmospheric plasma was evaluated based on the zones of inhibition and examined in terms of the enterococcal group and the “degree” of drug resistance.

**Results:**

Cold atmospheric plasma treatment killed all groups. Comparison of VRE and HLGR strains with non-VRE and non-HLGR isolates concerning zones of inhibition revealed that enterococci with special resistance patterns (VRE and HLGR) showed significantly smaller zones of inhibition than the sensitive ones. The mean of all isolates, irrespective of belonging to groups, showed smaller zones of inhibition with increasing “degree” of drug resistance.

**Conclusions:**

Cold atmospheric plasma treatment killed all isolates of enterococci, but its efficacy depended on the “degree” of drug resistance and on membership in special resistance groups with particular clinical-outbreak importance. However, a possible role of the different genetic lineages, which might be prone to acquiring more or less resistance phenotypes, may also play a role in this context.

**Electronic supplementary material:**

The online version of this article (doi:10.1186/s13099-016-0122-4) contains supplementary material, which is available to authorized users.

## Background

The microorganisms most often involved in multi-drug (MDR) resistance are the so-called ESKAPE pathogens (*Enterococcus faecium*, *Staphylococcus aureus*, *Klebsiella pneumoniae*, *Acinetobacter baumannii*, *Pseudomonas aeruginosa*, and enterobacteriaceae except *Klebsiella*), suggesting their proclivity in escaping antibacterial treatments [1, 2]. Beyond the search for new antibiotic drugs, novel antimicrobial strategies as alternatives to conventional agents with antimicrobial efficacy are strongly needed [3]. One new concept is cold atmospheric plasma (CAP), which combines potent physical properties such as ultraviolet and infrared radiation (within the near infrared region), reactive oxidative species, and charged particles [4–7]. In previous studies, the present authors showed significant efficacy of CAP in vitro against bacterial and fungal species [5, 8]. Successful treatments of different infectious diseases based on the considerable antimicrobial efficacy of CAP have been documented; hence, it can be deduced that plasma could be effective in hospital hygiene and wound medicine, as chronic wounds represent an important risk factor for MDR colonization [9–16].

However, to fulfil aforementioned high expectations in CAP as innovative alternative in the treatment of MDR pathogens, CAP efficacy should not be affected by resistance to antibiotics.

Therefore, two factors possibly influencing plasma susceptibility of enterococci were investigated: first, the “degree” of multi-drug resistance of enterococci, and second, the role of pathogens known as outbreak strains—the vancomycin-resistant enterococci (VRE) and/or enterococci resistant to high doses of gentamicin (HLGR).

## Methods

The aim of this in vitro-study was to determine the susceptibility of clinical isolates of enterococci showing different patterns of antimicrobial resistance to CAP treatment.

### Plasma source

The plasma source used was a dielectric barrier discharge (DBD) plasma device with a high voltage electrode of 20 mm in diameter developed by the CINOGY GmbH, Duderstadt, Germany. The electrode is being covered with a dielectric barrier made of an industrial ceramic. The pulse repetition rate of the device was adjusted on 250 Hz which leads to electric power dissipated in the gas discharge within the range of 167–237 mW [17]. The area covered by the visible plasma beam averaged 314 mm^2^.

### Susceptibility test model in vitro

CAP susceptibility was calculated as described detailed in a previous publication [5]. In few words, before starting plasma treatment, the strains were thawed and cultured aerobically at 36 °C overnight on sheep blood agar (Columbia agar, Biomérieux, Nürtingen, Germany). Suspensions were made out of these overnight cultures, diluted in sterile saline (1 colony in 1 ml). This suspension was diluted 1/100 in sterile saline (optical density was proved by measurement of McFarland) and 100 ml were plated on Columbia blood agar under sterile conditions. All isolates of enterococci were treated for 3 s by CAP on agar. Treatments were done under controlled room climate with temperatures not surpassing 21 °C and a humidity of ca. 45 %.

To compute the CAP susceptibility, the diameters (mean of two measurements at a 90° angle) of the obtained circular zones of inhibition (ZOI) were measured and the area calculated.

### Microbiology

A total of 39 clinical isolates were included, 16 *E. faecium* and 23 *Enterococcus faecalis*. Isolates were recovered consecutively during routine wound microbiology at the University Hospital of Greifswald, Germany. VRE identified as outbreak strains and isolated elsewhere were provided by the National Reference Center for Staphylococci and Enterococci, Robert Koch Institute, Wernigerode, Germany [18].

The different isolates were grouped according to their resistance pattern, including sensitive, vancomycin resistance (VRE) and high level gentamycin resistance (HLGR) as seen in Table 1 (membership of one isolate in several groups was possible).

**Table 1:** Total number (n) of isolates and number of specific species within the different groups

	Group 1 other enterococci (oE)^b^	Group 2 HLGR only	Group 3^a^ group 2 + 4	Group 4 VRE and HLGR	Group 5^a^ group 6 + 4	Group 6 VRE only
n of isolates	12	11	19	8	16	8
*E. faecium*	1	5	9	4	10	6
*E. faecalis*	11	6	10	4	6	2

^a^Group 3: containing all HLGR strains, group 5: containing all VRE strains

^b^Enterococci with susceptibility to vancomycin and high concentrations of gentamicin

**Fig. 1:**
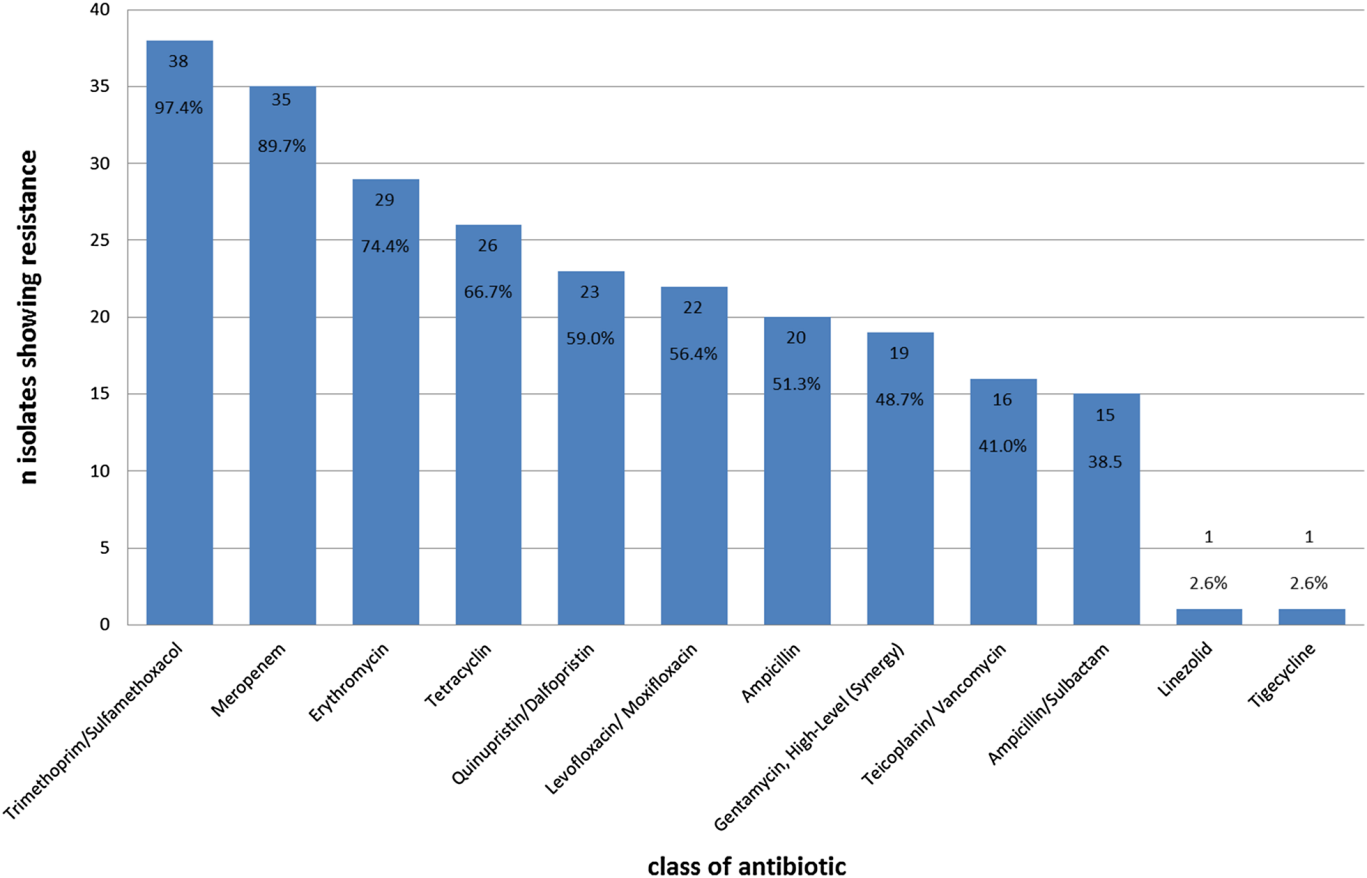
Number (n) of isolates showing resistance to the respective class of antibiotics and related percentage

Irrespective of above-mentioned resistance patterns were all 39 isolates differentiated into subgroups depending on their “degree” of antimicrobial resistance (resistance group, RG), due to the automated break-point determination test (breakpoints based on DIN and CA-SFM as interpretation criteria) in the VITEK® 2 Compact System (bioMérieux, Nürtigen, Germany). The 12 classes of antibiotic agents tested are given in Fig. 1 with the corresponding percentage of isolates showing resistance. The maximum number of resistances of one isolate in this study was 10, meaning that this isolate was placed in RG10.

### Statistical analysis

A paired t test with a significance level of 5 % was applied to compare the species-specific (groups 1–6) ZOI obtained after plasma treatment. Furthermore, an analysis of variance was performed to test for significant differences related to the “degree” of resistance (RG 2–10). For all calculations, the free software package R (R Development Core Team, R Foundation for Statistical Computing, Vienna, Austria) was used.

## Results

In all enterococci isolates plasma treatment resulted in circular bacteria-free areas (ZOI) in the homogeneous growth zone of the agar surface within 48 h.

**Fig. 2:**
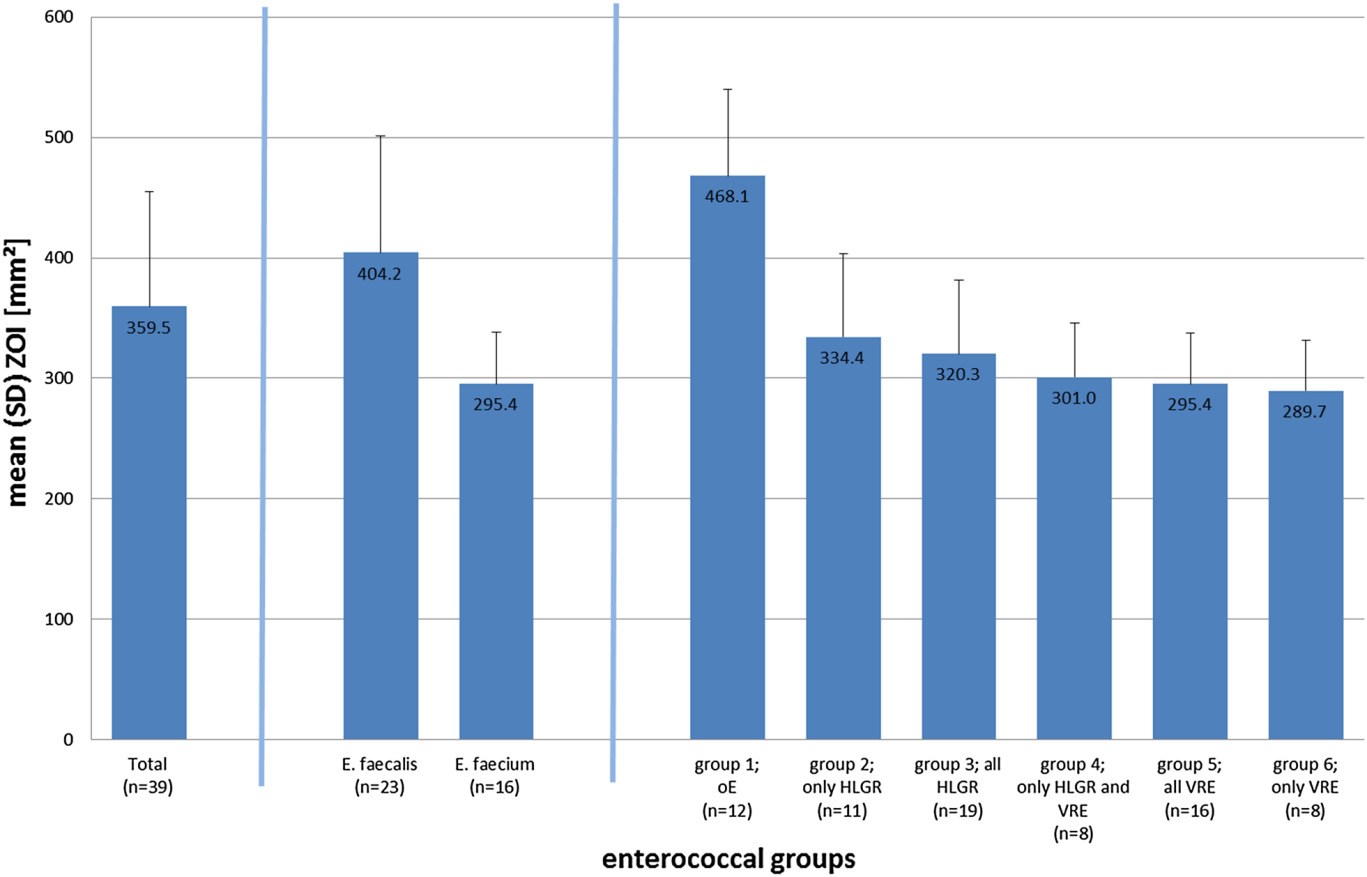
Mean (ranked) zone of inhibition (ZOI) (mm^2^ with SD) on agar obtained after treatment with DBD, total and by group

The ZOI of the enterococcal groups 1–6 are presented in Fig. 2. The group of “other enterococci” (group 1) showed the biggest ZOI (486 mm^2^). The VRE (group 6) exhibited the smallest ZOI (290 mm^2^). The difference between the maximum (oE, group 1) and minimum ZOI (only VRE, group 6) measured 178.4 mm^2^, equal to 38.1 %. Additionally, *E. faecium* exhibited higher CAP resistance than *E. faecalis*.

The comparison of groups and pooled groups of enterococcal isolates with different antimicrobial resistance patterns (i.e. VRE, HLGR, etc.) revealed significant differences in sizes of ZOI (Table 2). Significantly smaller ZOI were found in VRE strains (group 5) as well as in HLGR isolates (group 3) compared with non-VRE and non-HLGR isolates, respectively. As shown by the significantly larger ZOI in group 1 compared with all other groups, the highest CAP efficacy was obtained in the group of enterococci without the clinically important resistance patterns (group 1).

**Table 2:** Significant differences (p value) in ZOI between the different enterococcal groups

Compared groups	Mean (SD) ZOI (mm^2^) of respective group	p value
Group 1 vs. 2	468.1 (71.6) vs. 334.4 (69.1)	0.000
Group 1 vs. 4	468.1 (71.6) vs. 301.0 (44.8)	0.000
Group 4 vs. 6	301.0 (44.8) vs. 289.7 (42)	0.000
Group 1 vs. all other isolates	468.1 (71.6) vs. 311.3 (56.0)	0.000
Group 3 vs. all other isolates	320.3 (61.0) vs. 396.8 (105.3)	0.017
Group 4 vs. all other isolates	301.0 (44.8) vs. 374.6 (97.7)	0.014
Group 5 vs. all other isolates	295.4 (42.4) vs. 404.2 (94.8)	0.000
Group 6 vs. all other isolates	289.7 (42.0) vs. 377.6 (95.7)	0.002

**Fig. 3:**
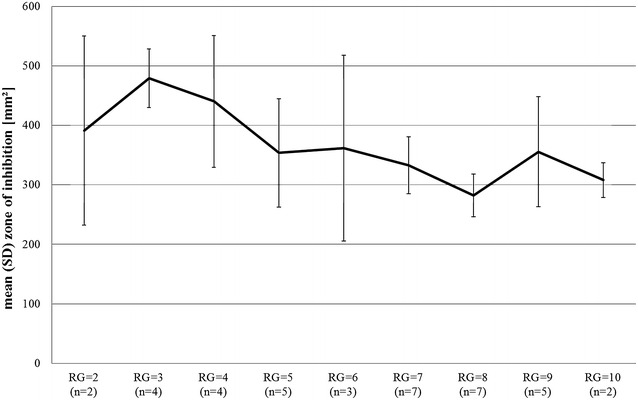
Mean (SD) ZOI (mm^2^) on agar obtained after treatment with DBD and respective resistance group (RG) of all enterococci

**Fig. 4:**
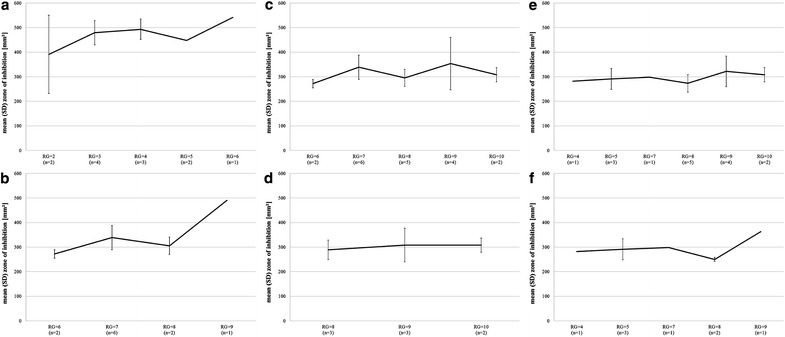
**a- f** Mean (SD) ZOI (mm^2^) on agar obtained after treatment with DBD and respective resistance group (RG) shown separately for group 1 (**a**), 2 (**b**), 3 (**c**), 4 (**d**), 5 (**e**) and 6 (**f**)

The statistical analysis of single isolates regarding the “degree” of drug resistance revealed that isolates with superior RG numbers showed smaller ZOI (p = 0.045) than isolates with lower RG numbers (Fig. 3). These findings did not occur within the statistical analysis of the enterococcal (resistance pattern) groups, probably due to the very small numbers within the different groups (Fig. 4a–f).

## Discussion

Clinically relevant enterococci primarily include two species, *E. faecalis* and *E. faecium*. Both species exhibit high levels of resistance against the aminoglycosides, and *E. faecium* is inherently resistant to β-lactam antibiotics. Currently, most isolates of *E. faecium* are highly resistant to ampicillin, while a non-negligible proportion exhibit high-level resistance to aminoglycosides and are resistant to glycopeptides, causing outbreaks in clinical settings [19].

Additionally, an increasing resistance to the newer antimicrobials linezolid, daptomycin and tigecycline could be observed in many countries [20].

Few studies have been published on plasma susceptibility of multidrug-resistant strains (e.g. VRE), such as those referring to decontamination of surfaces in hospital environments [21, 22]. However, no data on enterococci in terms of antibiotic resistance are available. From previous studies focussing on basic plasma suitability for inactivating wound and skin bacteria [8, 20, 23], no statistically supported conclusions on enterococcal susceptibility to plasma treatment can be drawn. Therefore, the antimicrobial efficacy of plasma from a well-researched plasma source (DBD) against a set of clinical isolates of enterococci, including VRE and HLGR, was investigated in vitro.

It was clearly demonstrated that the DBD easily eliminated all isolates of enterococci examined here, regardless of special resistance patterns such as VRE and HLGR. Interestingly, significant differences were found when the obtained ZOI diameters of distinct groups were compared.

The most obvious finding was that *E. faecium* was more resistant to plasma treatment than *E. faecalis*, which resembles the well-known greater drug susceptibility of *E. faecalis* compared to that of *E. faecium*. The reasons for the difference in plasma susceptibility of these two species could not get cleared in this study as the number of isolates did not allow deeper statistical analysis. Nevertheless, this result could be the next hint to related resistance mechanisms to CAP and antibiotics in enterococci.

The next important observation is that the authors found lower CAP susceptibility of VRE and HLGR treated with DBD in comparison with enterococci lacking these clinically important resistance patterns.

In addition, “higher” resistance to chemical antibiotics was correlated with less susceptibility to DBD (decrease in killing-zone diameter). This correlation was found only for all isolates irrespective of belonging to specific resistance groups probably due to the very small numbers within the different groups.

Based on the overall results, an influence of vancomycin resistance and HLGR on CAP susceptibility can be assumed for DBD, since the properties “vancomycin resistance” and “HLGR alone” confer significantly smaller ZOI on agar. However, a possible influence of the different genetic lineages, which might be prone of acquiring more or less resistant phenotypes, may also play a role in this context.

Although the data presented here strongly indicate plasma susceptibility of all enterococci, the lower susceptibility of *E. faecium* (compared to *E. faecalis*), VRE, HLGR, and generally MDR enterococci against DBD suggest the need for further research to clarify the as-yet unknown mechanisms by which bacteria are killed by CAP. This phenomenon could also help recognize and manage potential bacterial resistance mechanisms, which have not yet been documented.

## Conclusion

Plasma treatment with DBD was followed by a significant reduction of all tested enterococci, irrespective of species and antibiotic resistance pattern. However, the obtained data indicate significantly less susceptibility of VRE and HLGR enterococci as well as less susceptibility of *E. faecium* to CAP derived from a DBD source and a correlation between decreasing susceptibility to CAP and increasing resistance level of enterococci (Additional file 1).

## Additional file

10.1186/s13099-016-0122-4 Each strain is given with their corresponding zone of inhibition and their detailed profile of antibiotic resistance.

## Abbreviations

CAP: cold atmospheric plasma
DBD: dielectric barrier discharge
HLGR: high-level resistance to gentamicin
MDR: multi-drug resistant
oE: other enterococci
RG: resistance group
SD: standard deviation
VRE: vancomycin resistant enterococci
XDR: extreme drug resistant
ZOI: zones of inhibition
